# Squalene synthase cloning and functional identification in wintersweet plant (*Chimonanthus zhejiangensis*)

**DOI:** 10.1186/s40529-018-0246-6

**Published:** 2018-12-11

**Authors:** Guanhua Liu, Jianyu Fu

**Affiliations:** 1 Tea Research Institute, Chinese Academy of Agricultural Sciences, Hangzhou, 310008 People’s Republic of China; 2 Key Laboratory of Tea Quality and Safety Control, Ministry of Agriculture and Rural Affairs, Hangzhou, 310008 People’s Republic of China; 3 Graduate School of Chinese Academy of Agricultural Sciences, Beijing, 100081 People’s Republic of China

**Keywords:** Wintersweet, Squalene, Squalene synthase

## Abstract

**Background:**

Three species of wintersweets: *Chimonanthus salicifolius* S. Y. Hu, *Chimonanthus zhejiangensis* M. C. Liu and *Chimonanthus grammalus* M. C. Liu are widely distributed in China. The three wintersweets belonging to the genus of *Chimonanthus* that can synthesize abundant terpenoids that are beneficial to human health. Their buds and leaves are traditional Chinese herb applied by the ‘She’ ethnic minority in southeast of China. Squalene is a multi-functional and ubiquitous triterpene in plants, which is biosynthesized by squalene synthase (SQS) using farnesyl diphosphate (FPP) as the substrate. The synthesis of squalene in wintersweet was not clearly. This work would provide us much help to further understand the terpene metabolism in wintersweet and its health function to people at phytochemistry and molecular levels.

**Results:**

In this study, we identified squalene component in the extractions of leaves of three wintersweets and isolated SQS genes from leaf transcriptomes. The three SQSs were highly conservative, so CzSQS from *C. zhejiangensis* was just determined the enzymatic activity. The in vitro expressed CzSQS that deleted two transmembrane domains could catalyze FPP to generate squalene with the presence of NADPH and Mg^2+^.

**Conclusions:**

The squalene was one of wintersweet leaves phytochemicals. The squalene synthases of three wintersweet plants were highly conserved. The CzSQS was capable to catalyze two FPP molecules to squalene.

**Electronic supplementary material:**

The online version of this article (10.1186/s40529-018-0246-6) contains supplementary material, which is available to authorized users.

## Introduction

Wintersweet (*Calycanthaceae*) contains ten species belonging to three genera: *Calycanthus* L. contains three species distributed in America and Eastern Asia, *Chimonanthus* contains six species originated in China, and *Idiospermum Blake* contains a single species distributed in Australia (Christenhusz and Byng 2016). The six species of *Chimonanthus* are further classified into aromatic type and nonaromatic type according to whether their leaves release aroma or not. (Zhang and Shen 1999). Three species of *Chimonanthus salicifolius* S. Y. Hu, *Chimonanthus Zhejiangensis* M. C. Liu and *Chimonanthus grammalus* M. C. Liu are aromatic type (Zhang and Shen 1999). *C. salicifolius* S. Y. Hu, an endemic species of China, has been used as Chinese traditional medicine to therapy cough, vomiting, heatstroke, rheumatic arthritis and measles for thousands of years (Ma et al. 2017). *C. zhejiangensis* M. C. Liu, only found in Zhejiang, Jiangxi and Fujian provinces, is also a Chinese herb and a courtyard ornamental plant, which is widely planted in the scenic area of Zhejiang province (Ouyang and Mai 2010). *C. grammalus* M. C. Liu is an ancient angiosperm and a transitional species between *Chimonanthus* and *Neochimonanthus*, and is an endangered and national-protected medicinal plant in China and mainly distributed in Anyuan and Huichang counties of Jiangxi Province (Jiang et al. 2015a, b). The three *Chimonanthus* plants mostly distributed in the area where ‘She’ ethnic minority lived in, so the tender stems were firstly applied by them as a traditional herb to treat cough and rheumatism for a long time, and the Chinese name of the galenical is Shiliangcha (herb tea) (Ma et al. 2017). To date, the three species of *Chimonanthus* have been made into herbal beverage named as ‘Gold tea’, a type of healthy decoction in the counties around Zhejiang, Fujian and Jiangxi provinces.

In order to identify the bioactive components, both volatiles and non-volatiles of wintersweet plants were taken into account. The non-volatiles of *Calycanthaceae* include alkaloid (Ma et al. 2015a, b), terpene (Li et al. 2016), flavone (Zhang et al. 2017), coumarin (Li et al. 2013; Wang et al. 2016) and so forth. The volatiles of wintersweet plants were essential oil, which can be used as cosmetics, perfumery and aromatherapy. The aromatic type species of *Chimonanthus* usually release large quantity of volatiles and abundant terpenes (terpenoids) as β-caryophyllene, β-elemene, γ-elemene, germacrene-D, trans-β-ocimene, sabinene, δ-cadinene were found in essential oils (Lv et al. 2012; Farsam et al. 2007). Phytochemical analyses have revealed that they were not only rich in essential oils but also in semi-volatile fractions. Recently, squalene was identified in the volatile oil of *C. salicifolius* by GC–MS (Zhou et al. 2013). We also found squalene in the extractions of leaves of *C. zhejiangensis* detected by GC × GC-TOFMS method in this work. Squalene, a triterpene with a formula of C_30_H_50_ including six isoprene units, is an important intermediate of the endogenous synthesis of cholesterol in animals or sterol in plants. It is ubiquitous and has been detected in bacteria, alga and mammals. In 1935, the first higher plant-derived squalene was gained from olive oil (Thorbjarnarson and Drummond 1935). Up to now, squalene has been isolated in more plants as *Arabidopsis thaliana*, *Vitis vinifera* (Nakashima et al. 1995; Kribii et al. 1997; Genova et al. 2014; Uchida et al. 2015).

Terpene or terpenoid is the largest class of secondary metabolites in plants, and it is synthesized by either the mevalonate pathway (MVA) in the cytosol or the methylerythritol phosphate pathway (MEP) in plastids (Jiang et al. 2015a, b). Squalene is synthesized in the triterpene biosynthesis pathway, a branch of the terpene biosynthetic network of MVA pathway in plants (Chappell et al. 1995, 2002). It is generated from two molecules of farnesyl diphosphate (FPP) in a two-step reductive dimerization reaction catalyzed by squalene synthase (SQS) (Pandit et al. 2000). The next metabolism step is that squalene is cyclized to lanosterol followed by many enzymatic steps for the conversion to cholesterol (Smith 2000). SQS is the key enzyme of squalene metabolism, and the genes have been isolated in animals, plants and bacteria (Pandit et al. 2000; Jiang et al. 2015a, b; Dale et al. 2013; Okada et al. 2000). SQSs from eukaryotic organisms are associated with the ER (endoplasmic reticulum) membranes through a hydrophobic membrane-spanning α-helix located at the C-terminus of the protein. In contrast, bacterial SQSs lack of the C-terminal α-helix, and are not affiliated with the membrane (Stamellos et al. 1993a, b). The SQSs have been studied deeply in many organisms including *Arabidopsis thaliana*, tobacco and human. In contrast, squalene was found in *C. zhejiangensis*, a traditional Chinese herb, but our knowledge on the molecular basis of squalene biosynthesis in which is absent. Here, we further confirmed squalene generation in the leaves of wintersweet, isolated the squalene synthase gene of *C. zhejiangensis* and identified its enzymatic activity, to give a complete explanation on the squalene biosynthesis in wintersweet.

## Methods

### Plant materials

Three species of wintersweet: *Chimonanthus salicifolius* S. Y. Hu, *Chimonanthus Zhejiangensis* M. C. Liu and *Chimonanthus grammalus* M. C. Liu were collected from Yushan and Wuyuan, Jiangxi province and Kaihua, Zhejiang Province. The leaves were stored at − 80 °C after being frozen in liquid N_2_.

### Squalene collection and detection in *C. zhejiangensis* leaves

Fresh leaves of *C. zhejiangensis* (3 g) was cut into pieces and sealed in a petri plate and then SPME (solid-phase micro-extraction, SUPELCO, Sigma-Aldrich Co.) column was injected into the plate to collect the volatiles. For more accurate analysis, the volatiles emitted from *C. zhejiangensis* leaves pieces were collected in an open headspace sampling system (Analytical Research System, Gainesville, FL) as previously reported (Li et al. 2012). Volatiles were collected for 24 h by pumping air from the chamber through a Super-Q volatile collection trap and eluted using 300 μL n-hexane containing 0.003% (w/v) 1-octanol as an internal standard. The leaves (1 g) was further ground into powder in liquid nitrogen and extracted by chromatographic *n*-hexane, dichloromethane and ethyl acetate at 37 °C and shaking at 200 rpm for 24 h. Finally, the extract was centrifuged at 12,000 rpm for 30 min and the supernatant was used for analyzing.

The non-volatile compounds of *C. zhejiangensis* were analyzed by GC × GC-TOFMS (multidimensional gas chromatography coupled to time-of-flight mass spectrometry LECO Pegasus 4D GC × GC-TOFMS instrument, LECO Corporation, St. Joseph, MI, USA) and Gas Chromatography-Mass Spectrometer (GC–MS, Agilent Technologies 7890B GC–MS system). The chromatographic column of GC × GC-TOFMS was Rxi-5MS column (30 m × 250 μm, Restek, Bellefonte, PA, USA) coupled to Rxi-17Sil MS column (1.9 m × 100 μm × 0.10 μm, Restek, Bellefonte, PA, USA). The GC–MS column was HP-5MS (30 m × 0.250 mm, 1909IS-433UI, Agilent Technologies. Inc. California, USA). For GC–MS analysis (Jiang et al. 2015a, b), the injection temperature was 250 °C and a temperature gradient of 5 °C/min from 50 °C (hold 3 min) to 250 °C. The electronic impact (EI) mode was at 70 eV. For GC × GC-TOFMS analysis (Yoshimi et al. 2018), samples were injected at 50 °C in the splitless mode. After holding the samples for 5 min at 50 °C, the oven temperature was increased at 10 °C/min to 300 °C and hold for 5 min. The flow rate of helium carrier gas was set at 1.9 mL/min. All MS data was collected from 40 to 400 m/z.

### Cloning of squalene synthase genes from wintersweet

The transcriptome of the *C. zhejiangensis* foliage was sequenced by Illumina. The data was assembled by the Trinity software (Grabherr et al. 2011) and the result was de-redundant and re-spliced by the Tgicl program to obtain the final unigene. The unigene was Blast with other organism SQSs by Blastp program. Total RNAs were extracted from leaves using Polysaccharide and Polyphenol Total RNA Isolation Kit (Tiangen Biotech Co., Ltd., Beijing, China). The quality and concentration were checked by NanoDrop 1000 spectrophotometer (Thermo Fisher Scientific, Waltham, MA, USA) and formaldehyde agarose gel electrophoresis. Total RNA (100 μg/μL) was reverse-transcribed into first strand cDNA in a reaction volume (80 μL) using PrimeScript™ RT reagent Kit with gDNA Eraser (Perfect Real Time) (TaKaRa, Co. Ltd, Dalian, China) following the manufacture’s protocol and diluted to 60 μL. The primers of 5′-ATGGGGAAGTTAGGAACAATGCTG-3′ and 5′-TCATTTAATGGAAAGAAATGCAAAC-3′ was designed based on the unigene sequence on primer 6.0. The PCR reaction was set as follows: pre-denature at 94 °C for 2 min; 35 cycles of denature at 98 °C for 10 s, annealing at 56 °C for 30 s, extension at 68 °C for 2 min; a final extension at 72 °C for 10 min. The PCR products were separated by agarose gel and purified using TIANgel Midi Purification kit (TIANGEN, Beijing, China), and then were cloned into pGEM-T Easy vector (Promega Corporation, Madison, WI, USA) to sequence bidirectionally with the universal primers of M13.

### Squalene synthase sequences analysis

The SQS genes from three species of wintersweet were translated into protein sequences by ExPASy online (http://web.expasy.org/translate/). Multiple sequences of other plant SQSs were retrieved by Blastp from NCBI (https://blast.ncbi.nlm.nih.gov/Blast.cgi), and the detail information of all SQSs was in Additional file 1: Table S3. Sequence alignment for SQSs was done by ClustalW program embedded in MAGE 7.0, and the phylogenetic tree was also constructed by MAGE 7.0 based on Neighbor Joining (NJ) method (Kumar et al. 2016). The protein structure model was constructed by Phyre2 Fold Recognition online (http://www.sbg.bio.ic.ac.uk/phyre2). The signal peptide and transmembrane region of SQSs were analyzed by SignalP 4.1 Server (http://www.cbs.dtu.dk/services/SignalP/) and TMHMM program online (http://www.cbs.dtu.dk/services/TMHMM/), respectively.

### Protein expression and enzymatic activity determination of SQS

The *C. zhejiangensis* squalene synthase (CzSQS) gene removed hydrophobic region was introduced into pEXP5-CT/TOPO vector and the recombinant plasmids were transformed into *E. coli* (BL21 Condon plus) by heat shock at 42 °C for 1 min. After the cells were rejuvenation in LB liquid medium at 37 °C, 225 rpm for an hour, they were cultured to an OD_600_ of 0.6 in 50 mL LB liquid medium with 50 μg/mL ampicillin and 25 μg/mL chloramphenicol at the same condition. Gene expression was induced by 1 mM IPTG (Isopropyl-β-d-thiogalactopyranoside, 50 mg/mL, TIANGEN, Ltd., Beijing, China) at 16 °C, 150 rpm for 20 h. The cells were harvested by centrifugation, sonicated for 6 × 30 s in chilled 1 × PBS buffer and desalted through Econo-Pac 10DG columns (Bio-Rad). The enzymatic activity was measured on the basis of the conversion of farnesyl diphosphate (FPP) (Echelon Biosciences) to squalene in the presence of NADPH and Mg^2+^: 250 μL of the reaction mixture included 10 mM MOPSO (pH 7.0), 25 μM FPP (Echelon Biosciences), 25 mM MgCl_2_, 25 mM mercaptoethanol, 5 mM NADPH and 125 μL enzyme. The reaction was conducted at 37 °C for 5 h. After incubation, the mixture was extracted three times with 500 μL *n*-hexane. The *n*-hexane solution was concentrated by the blowing of nitrogen at room temperature until the total volume reached approximately 120 μL (Jiang et al. 2015a, b). The catalytic product was analyzed by GC–MS (Agilent 7890B-7000C, Agilent Technologies. Inc. California, USA) and confirmed by authentic standard of squalene.

## Results

### Identification of squalene in leaves of wintersweet

**Fig. 1:**
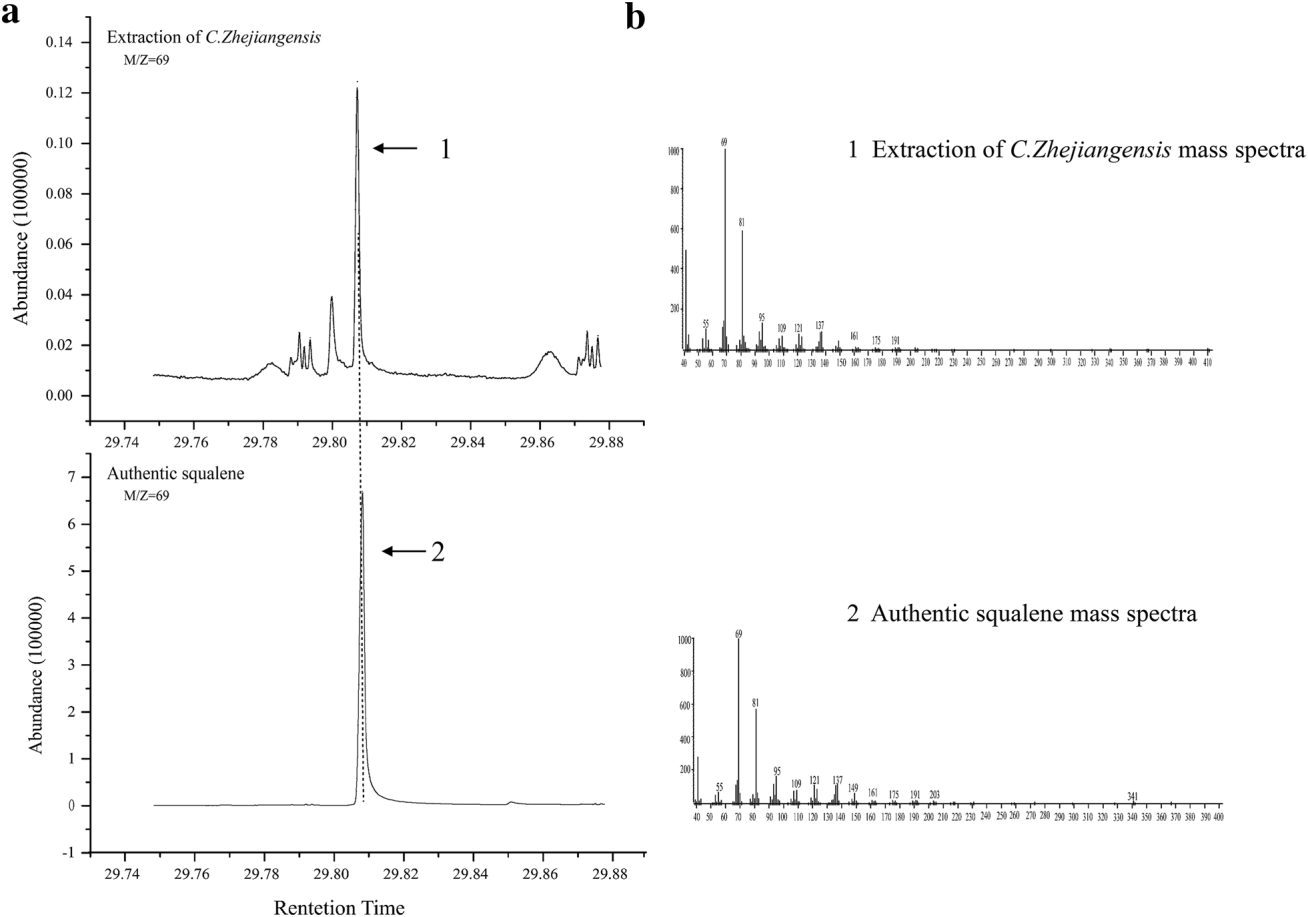
A partial gas spectrum of the *C. zhejiangensis* foliage extraction with dichloromethane

Squalene was not found in the volatiles of *C. zhejiangensis* leaves collected by the SPME and Super-Q using static and dynamic headspace methods, while it was detected by GC × GC-TOFMS in the leaf extractions of *n*-hexane, dichloromethane and ethyl acetate. Both the 1st and 2nd dimension time (minutes/s) and the unique mass were equivalent with the authentic standard of squalene (Fig. 1). The chromatogram peak area of the extractions of dichloromethane was the largest, which indicated that although squalene was identified in all the extractions of three solvent, the extraction efficiency of dichloromethane was the highest (Fig. 1).

### Isolation of squalene synthase genes from wintersweet

The full-length sequences of squalene synthase from *C. salicifolius, C. zhejiangensis*, *C. grammalus* were first identified by transcriptomic sequencing and further confirmed by PCR amplification and the NCBI accession numbers are MH277638, MH277639 and MH277637, respectively. Although the sequences of wintersweet plants were all 1227 bp long and encoded 408 amino acids, there were five different sites among the three SQSs (Additional file 1: Figure S1). At 42nd, 52nd and 313rd sites, the SQSs of *C. zhejiangensis*, *C. grammalus* and were Ile, Ser and Met, but the three sites were Thr, Gly and Leu in *C. Salicifolius* squalene synthase (CsSQS). At the positions of 172nd and 208th, the sequences of CsSQS, CzSQS were Val and Ala, while the same sites replaced by Ala and Thr in *C. grammalus* squalene synthase (CgSQS), respectively.

**Fig. 2:**
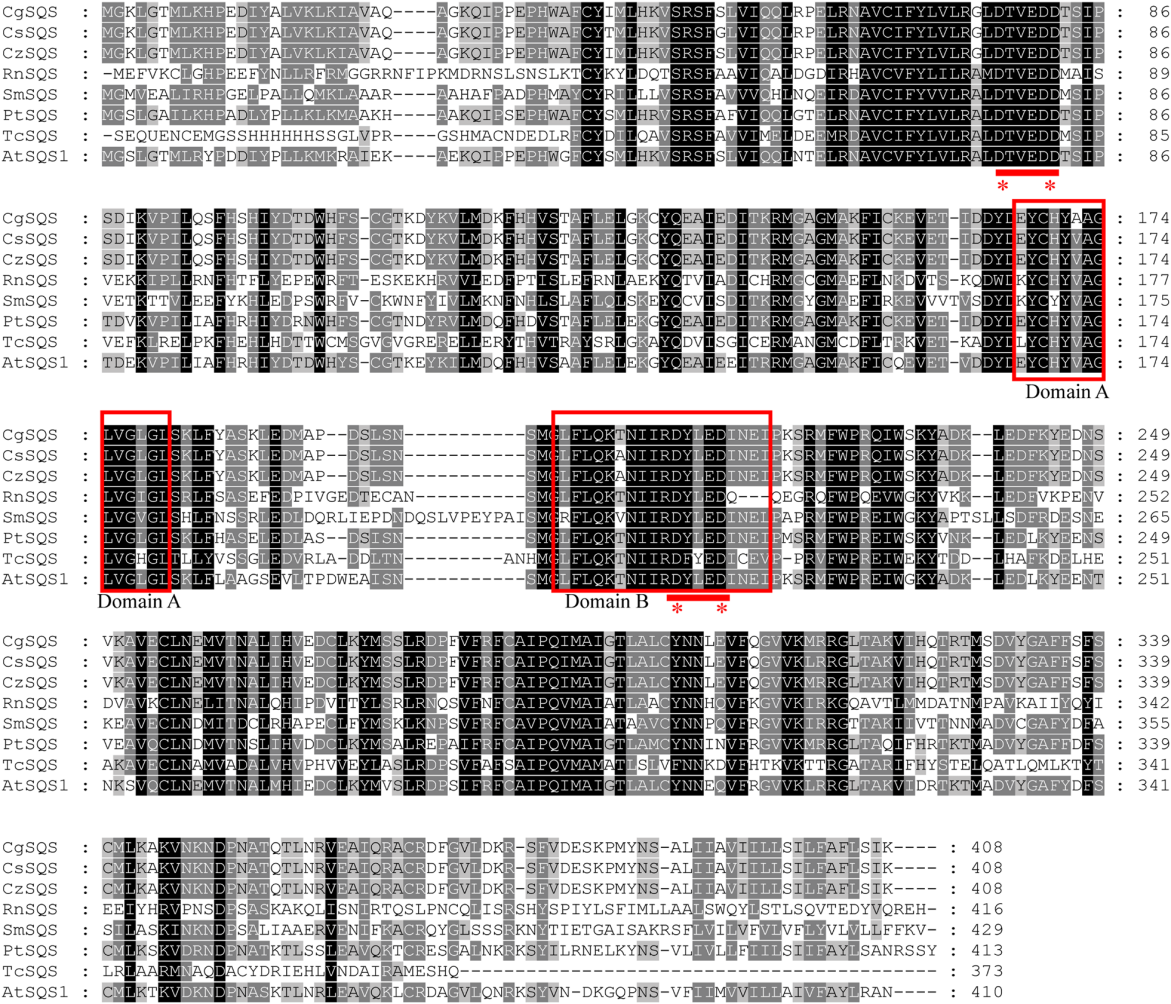
Multiple sequences alignment of SQSs from three wintersweet plants and other organisms: AtSQS1 (*Arabidopsis thaliana* SQS1), RnSQS (*Rattus norvegicus* SQS), SmSQS (*Selaginella moellendorffii* SQS), PtSQS (*Populus trichocarpa* SQS) and TcSQS (*Trypanosoma cruzi* SQS). The two conserved domains, DXXXDD and DXXXD, which mediate the binding prenyl diphosphate are underlined among SQSs from different species

Multiple sequences alignment was made by MAGE7.0 (Fig. 2) to further confirm the SQS sequences of three wintersweet plants. The sequences, including *Arabidopsis thaliana* SQS1 (Nakashima et al. 1995), *Rattus norvegicus* SQS (McKenzie et al. 1992), *Selaginella moellendorffii* SQS (Jiang et al. 2015a, b) and *Populus trichocarpa* SQS (Tuskan et al. 2006) were searched from the NCBI Blastp, and the function of these enzymes were conducted. The TcSQS (*Trypanosoma cruzi* SQS, Shang et al. 2014), elucidated senior structure and obtained on the Phyre2 on Internet, was included for comparison in this work. The two aspartate rich domains ‘DXXXDD’ and ‘DXXXD’ that mediate the binding of prenyl diphosphate are highly conserved among all the SQSs. The five different sites were out of the active domain compared with other SQSs.

### Homology-based structural modeling and phylogenetic analysis of SQSs

**Fig. 3:**
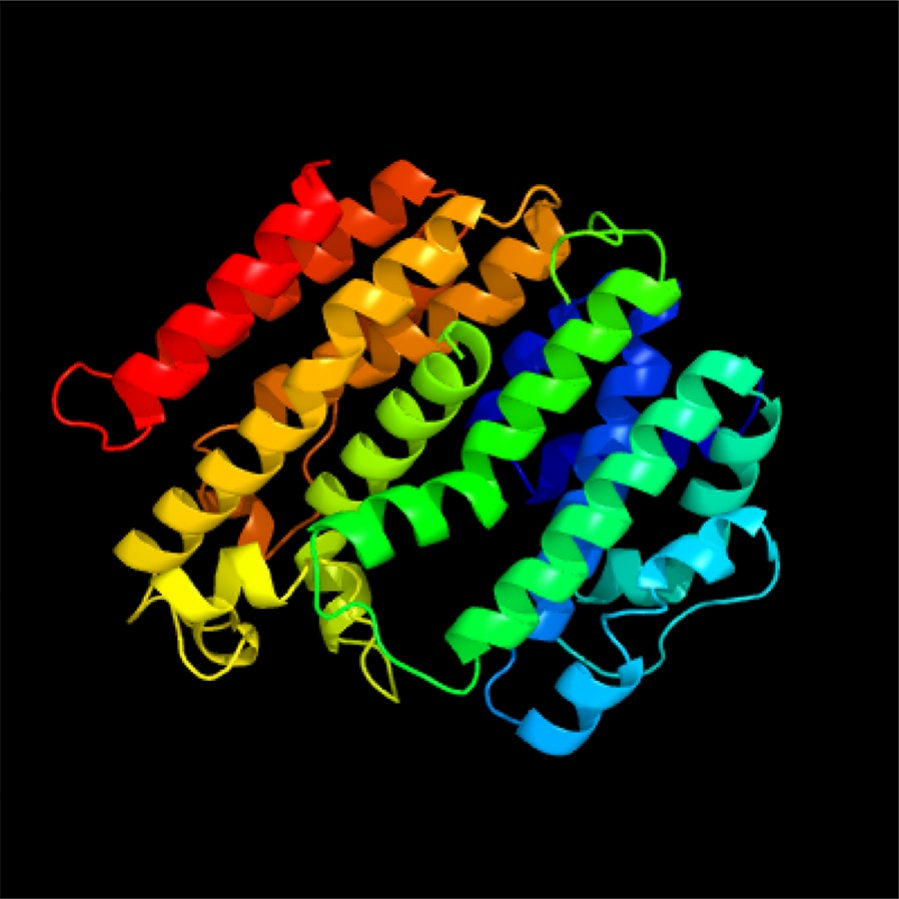
The homology-based structural modeling of c3wccC (TcSQS)

The homology-based structural modeling on Phyre2 indicated that the model of three wintersweet SQSs were similar to *Trypanosoma cruzi* squalene synthase (TcSQS, Fig. 3, Shang et al. 2014), and they were 100% confidence with TcSQS. The different amino acids of three wintersweet SQSs, located at the 42nd, 52nd, 172nd, 208th and 313th sites, which didn’t affect the active center structure formation of α-helix on the base of the homology-based structural modeling of TcSQS in the same sites. The analyses of TMHHM and SingalP showed that CsSQS, CzSQS and CgSQS contained no any N-terminal signal peptide but one C-terminal transmembrane region located at 390th to 407th amino acids (Additional file 1: Figure S2).

**Fig. 4:**
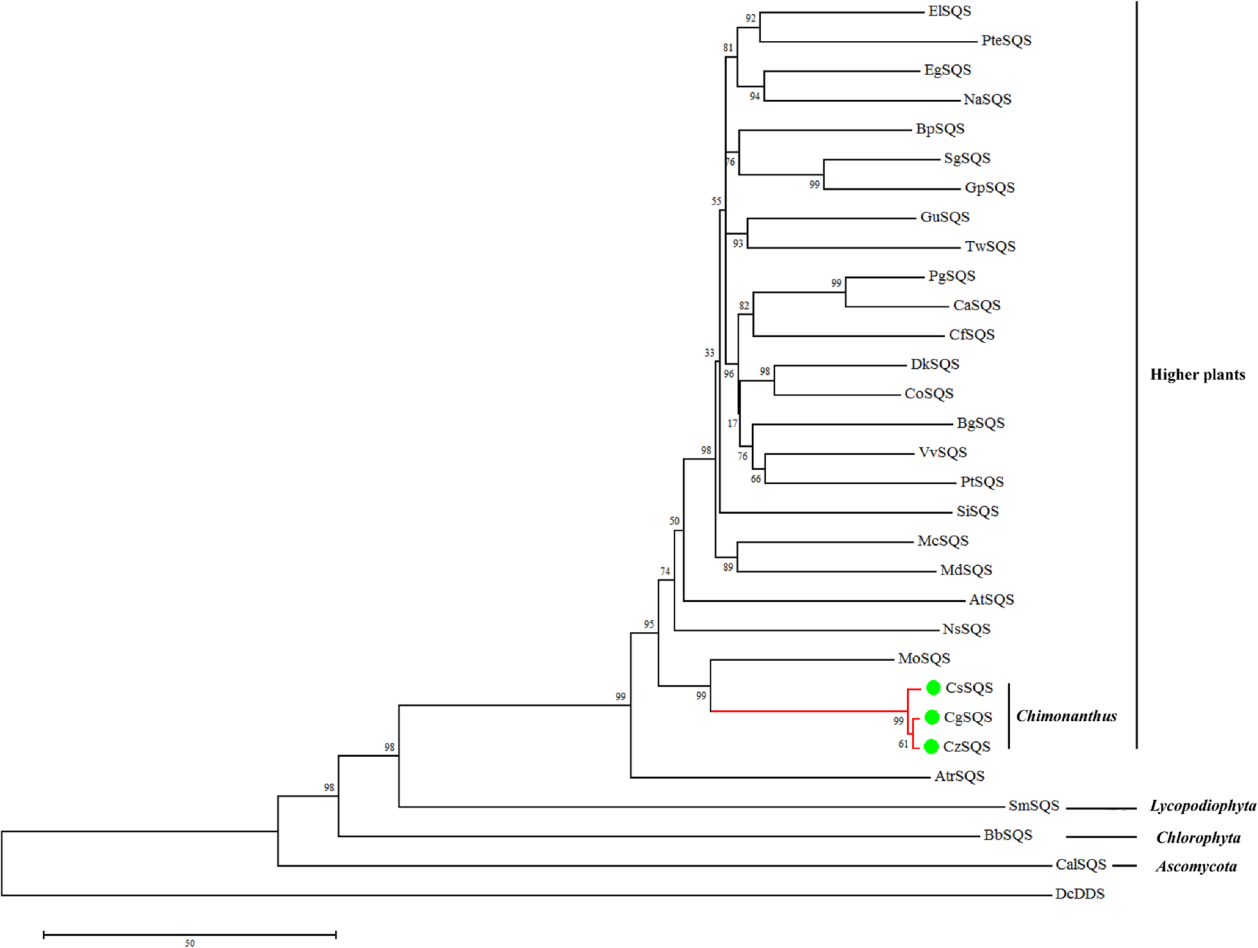
Phylogenetic tree of SQSs. The NCBI accession number of the sequences was in Additional file 1: Table S3

Other twenty-eight SQSs of fungi, alga, lichen and higher plants were retrieved from NCBI to run the phylogenetic analysis with three SQSs of wintersweet. The phylogenetic tree was divided into four large groups based on the four phyla: the SmSQS (Jiang et al. 2015a, b), BbSQS (Okada et al. 2000) and CalSQS (Dale et al. 2013) are on behalf of Lycopodiophyta, Chlorophyta and Ascomycota, and the rest SQSs clustered into another branch as the spemaiophyla (Fig. 4). The SQSs of three Chimonanthus plants clustered into one clade and then they prior clustered with MoSQS (*Magnolia officinalis* squalene synthases, Zha et al. 2016) (Fig. 4). It indicated that MoSQS had close relationship with the SQSs from wintersweet, which perhaps because both *Magnolia officinalis* and Chimonanthus were Magnoliids plants. The detail information of the SQSs is available in Additional file 1: Table S3.

### *E. coli* expressed SQS catalyzes the formation of squalene using FPP as substrate

**Fig. 5:**
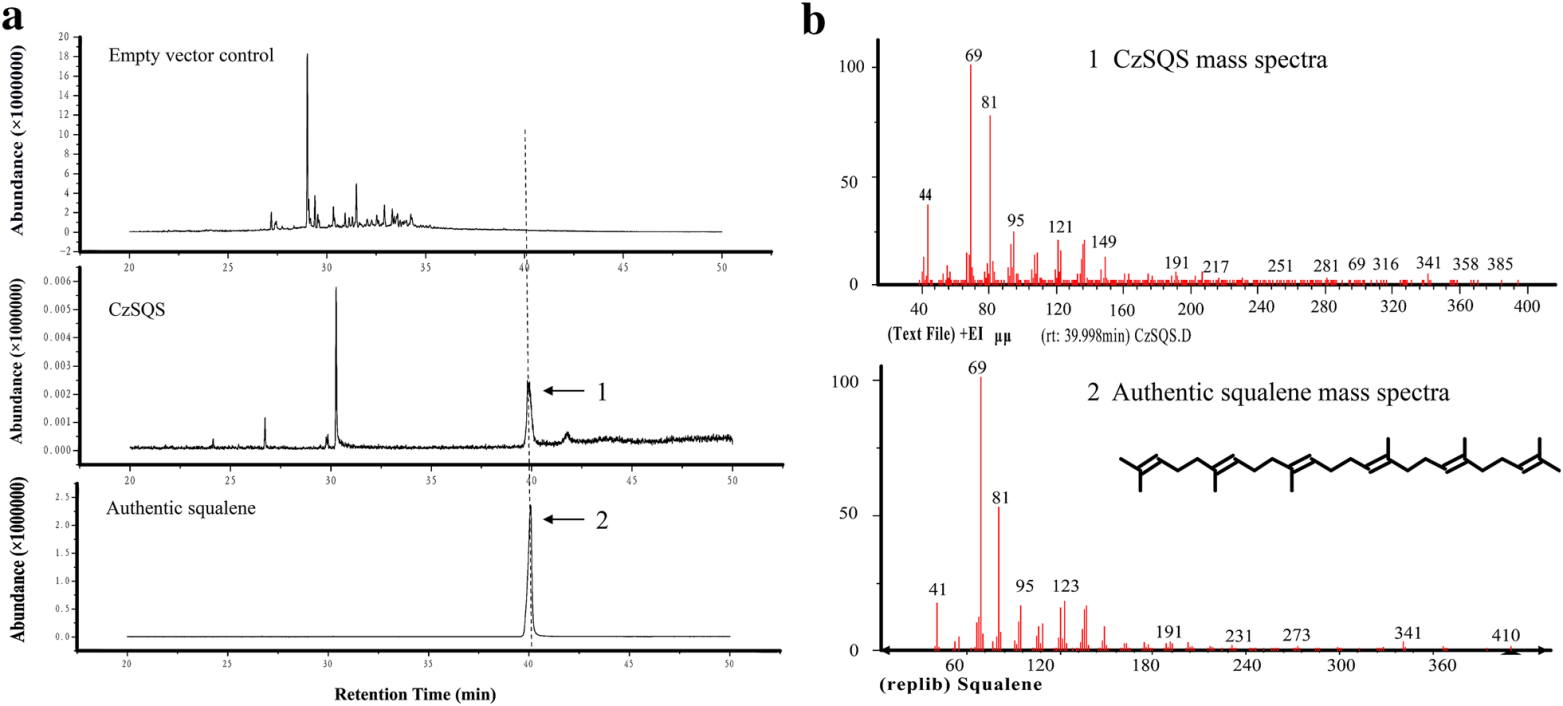
Partial gas chromatogram of products of squalene synthase enzyme assays. **a** Gas chromatogram of the empty vector control (pET32a), CzSQS reflects squalene synthase enzyme reaction catalyzed by recombinant CzSQS expressed in *E. coli* and the authentic squalene from SIGMA. **b** Mass spectrum of the 39.998-min peak in the gas chromatogram of the CzSQS and the authentic squalene at the same time

Eukaryotic squalene synthases localized in the ER membrane through its C-terminus, which usually lead to insoluble question when expression eukaryotic SQSs in *E. coli*. The deletion of residues from the C-terminus of tobacco SQS (Hanley et al. 1996; Devarenne et al. 2002), capsicum SQS (Lee et al. 2002) and yeast SQS (LoGrasso et al. 1993) resulted in functionally soluble enzymes. The coding sequence of CzSQS without C-terminal member-anchoring peptide was cloned and expressed in *E. coli* (strain: BL21 Condon plus) to determine the enzymatic activity. The catalyzed product in vitro was detected in GC–MS, and its peak at 39.998 min was the same as that of authentic squalene (Fig. 5a). Nevertheless, no any peak was detected at uniform retention time in the empty vector as control. The major mass fragments of m/z = 69 and m/z = 81 at 39.998 min of the peak of CzSQS is consistent with full-scan mass spectrum of authentic squalene (Fig. 5b). The results indicated that CzSQS encoded a functional SQS enzyme which converted two farnesyl diphosphate molecules into a squalene in vitro without the C-terminal member-anchoring peptide.

## Discussion

Wintersweet has attracted much attention not only because of their ornamentality and rarity, but also due to their affluent pharmacologically active substances. Their buds and leaves have been applied as a traditional herb by the ‘She’ ethnic minority people that distributed in the southeast of China. The pharmacology studies of wintersweet have revealed that its volatile or non-volatile compounds has phytochemical functions as anticonvulsion, abirritation, inhibiting cancer cells and anti-mycobacteria (Chebib et al. 2003; Amador et al. 2000; Verotta et al. 2002; Ma et al. 2015a, b; Wang et al. 2011; Yu et al. 2014). It was reported that 35.97% of the volatile compounds of wintersweet was sesquiterpenoids, and so the essential oil has anti-bacteria and free radical scavenging functions (Yu et al. 2014). In this paper, we analyzed the volatiles and non-volatiles compounds in leaves of wintersweet by SPME and GC × GC-TOFMS methods, respectively. Totally, 54 volatile terpenoids including 25 monoterpenoids and 29 sesquiterpenoids were detected in the leaves of *C. zhejiangensis* by SPME. The contents of eucalyptol, β-myrcene and pinene were the highest in monoterpenoids, and germacrene D, cadina-1(10), 4-diene and copaene were the highest in sesquiterpenoids. In the concentrated extractions of *n*-hexane, dichloromethane and ethyl acetate, up to 104 terpenes or terpenoids were identified and some of them were also included in the volatiles detected by the SPME (Additional file 1: Tables S1 and S2). Our result was similar to other studies that the terpenes or terpenoids were the major compounds of the wintersweet plants. Besides large quantity of monoterpenoids and sesquiterpenoids, we also found squalene in the extractions of leaves of *C. zhejiangensis* and which has been confirmed in one species of wintersweet (Zhou et al. 2013).

Squalene is taken part in the ergosterol biosynthetic process, sterol biosynthetic process and terpene metabolism. Squalene synthase (SQS), the farnesyl-diphosphate farnesyl transferase, catalyzes two molecules farnesyl diphosphate to form squalene. SQS is the key enzyme of the ergosterol, sterol and terpene metabolism. Here, we cloned SQSs from three wintersweet plants and identified the function in vitro for the first time. The subcellular location of AtSQS1 is the endoplasmic reticulum (ER) (Busquets et al. 2008). The CzSQS, CgSQS, CsSQS transmembrane regions were similar to AtSQS1 based on the sequences alignment and the TMHHM analysis (Additional file 1: Figure S2). The subcellular of the wintersweet SQS was like the AtSQS1, they locate on the ER. The active site of domain A, combining the two FPP into PSPP and loosen one pyrophosphate (Gu et al. 1998), is Try. These sites are the same among the three wintersweet plants SQS. The different five sites of the three wintersweet plants SQS was absent of the active site. In domain B, binding the diphosphate moiety of the allylic substrates via an Mg^2+^ bridge (Gu et al. 1998), the active domain is DXXXD that binding prenyl diphosphate. The different amino acid residues of the three wintersweet plants SQSs are absent in DXXXD. The other different sites were not in the active domain (Additional file 1: Figure S1).

In view of highly conserved sequences and homology-based structural modeling result, we just performed the enzymatic analysis for CzSQS. Prokaryotic expression demonstrated that CzSQS-pEXP5-CT/TOPO was expressed in its soluble form in *E.coli* (strain: *BL21 Condon plus*) induced with 1 mM IPTG. In vitro enzyme reactions manifested that CzSQS-pEXP5-CT/TOPO encoded a functional squalene synthase. Squalene is spotlighted by its biofunction in clinic usage, such as antioxidant, antitumor, and cyto-protective effects (Kim and Karadeniz 2012). While the function of squalene from wintersweet plants was deficiency. On the other hand, the botanical squalene was restricted to olive oil, camellia oleifera, amaranth and palm (Murkovica et al. 2004; Mlakar et al. 2010; Lehmann 1996; Sun et al. 1995). We isolated the squalene synthase and authenticated squalene in the leaves of *C. zhejiangensis*. The results provide a theoretical basis for the development and utilization of Shiliangcha, buds and leaves of the three wintersweet plants, and its pharmacological analysis.

## Conclusions

In a word, squalene was detected in the extraction of *C. zhejiangensis* leaves. The three subspecies wintersweet plants squalene synthase were cloned and analyzed results indicated that the three squalene synthase were highly conversed. The CzSQS possessed biological activity in catalyzed the squalene synthesis.

## Additional file

**Additional file 1: Figure S1.** Alignment of three wintersweet plants SQSs protein sequences. The black blocks are the locations of the different sites and the conserved domains was marked by the red underline. **Figure S2.** The TMHMM and SignalP prediction result about the three wintersweet SQSs and the SQS1 of *Arabidopsis thaliana*. **Table S1.** The compounds of dichloromethane extraction of *C. Zhejiangensis* foliage. **Table S2.** The volatiles compounds of *C. Zhejiangensis* foliage by SPME. **Table S3.** The accession number of SQSs sequences.

## Abbreviations

CzSQS: *Chimonanthus zhejiangensis* squalene synthase
CsSQS: *Chimonanthus salicifolius* squalene synthase
CgSQS: *Chimonanthus grammalus* squalene synthase
TcSQS: *Trypanosoma cruzi* squalene synthase
