# Supplementary material for: Squalene synthase cloning and functional identification in wintersweet plant (Chimonanthus zhejiangensis)
Source: Bot Stud. 2018 Dec 11;59:30. doi: 10.1186/s40529-018-0246-6 (PMC6289936; doi:10.1186/s40529-018-0246-6)
Supplement: Supplementary file 1 — Additional file 1: Figure S1. Alignment of three wintersweet plants SQSs protein sequences. The black blocks are the locations of the different sites and the conserved domains was marked by the red underline. Figure S2. The TMHMM and SignalP prediction result about the three wintersweet SQSs and the SQS1 of Arabidopsis thaliana. Table S1. The compounds of dichloromethane extraction of C. Zhejiangensis foliage. Table S2. The volatiles compounds of C. Zhejiangensis foliage by SPME. Table S3. The accession number of SQSs sequences. [file 40529_2018_246_MOESM1_ESM.pptx]

## Slide 1
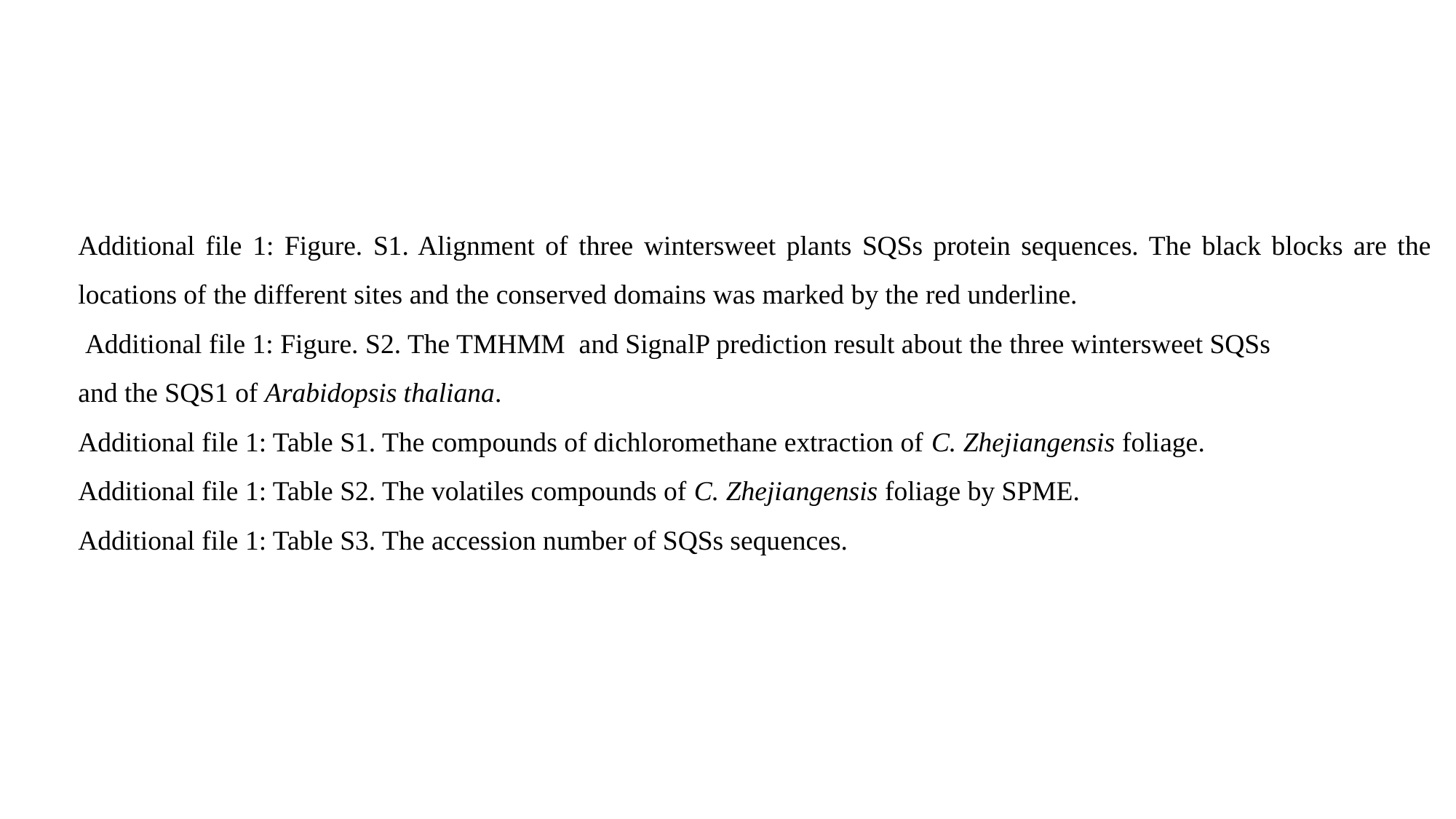

Additional file 1: Figure. S1. Alignment of three wintersweet plants SQSs protein sequences. The black blocks are the locations of the different sites and the conserved domains was marked by the red underline.
 Additional file 1: Figure. S2. The TMHMM and SignalP prediction result about the three wintersweet SQSs
and the SQS1 of Arabidopsis thaliana.
Additional file 1: Table S1. The compounds of dichloromethane extraction of C. Zhejiangensis foliage.
Additional file 1: Table S2. The volatiles compounds of C. Zhejiangensis foliage by SPME.
Additional file 1: Table S3. The accession number of SQSs sequences.

## Slide 2
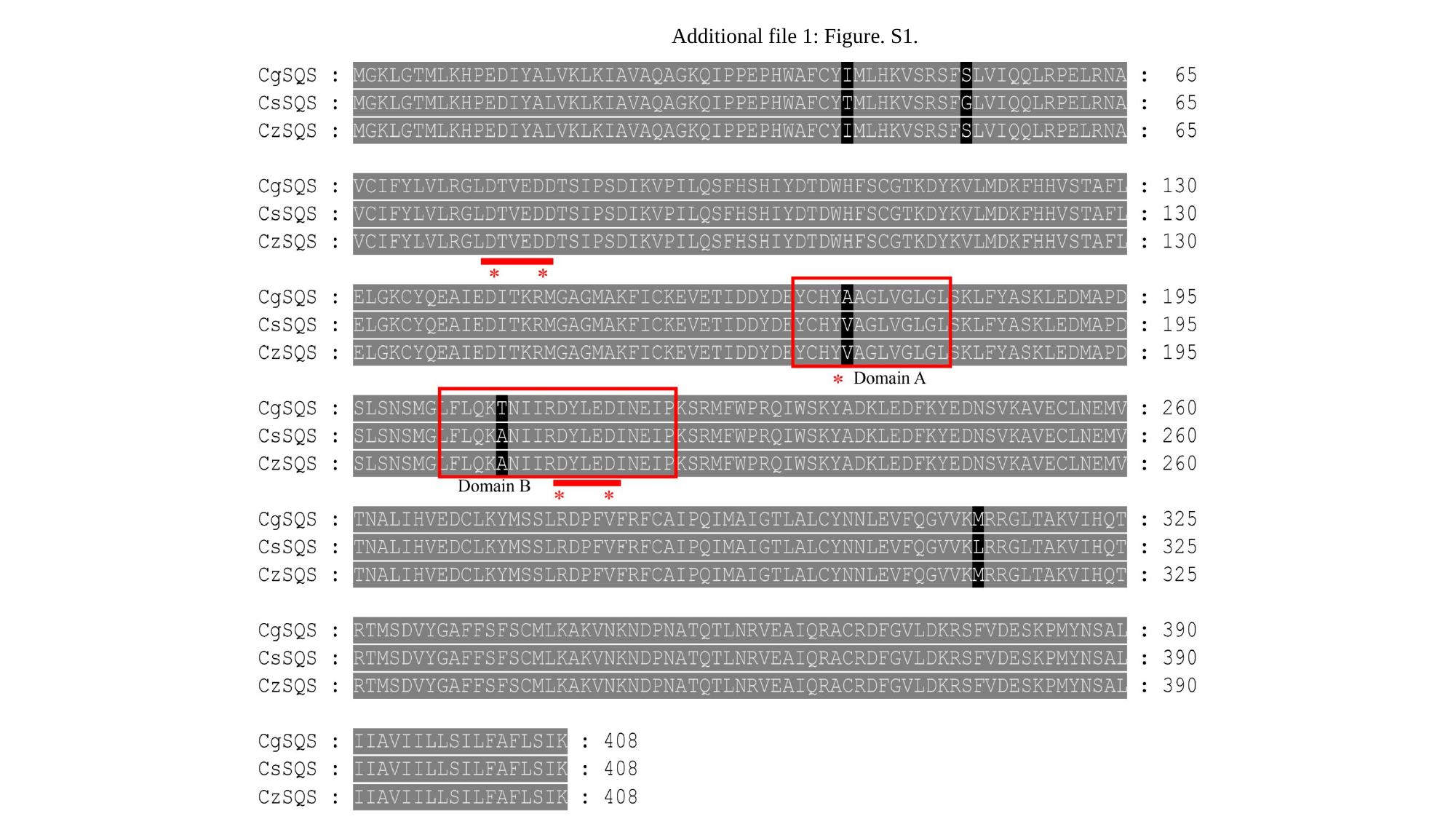

Additional file 1: Figure. S1.

## Slide 3
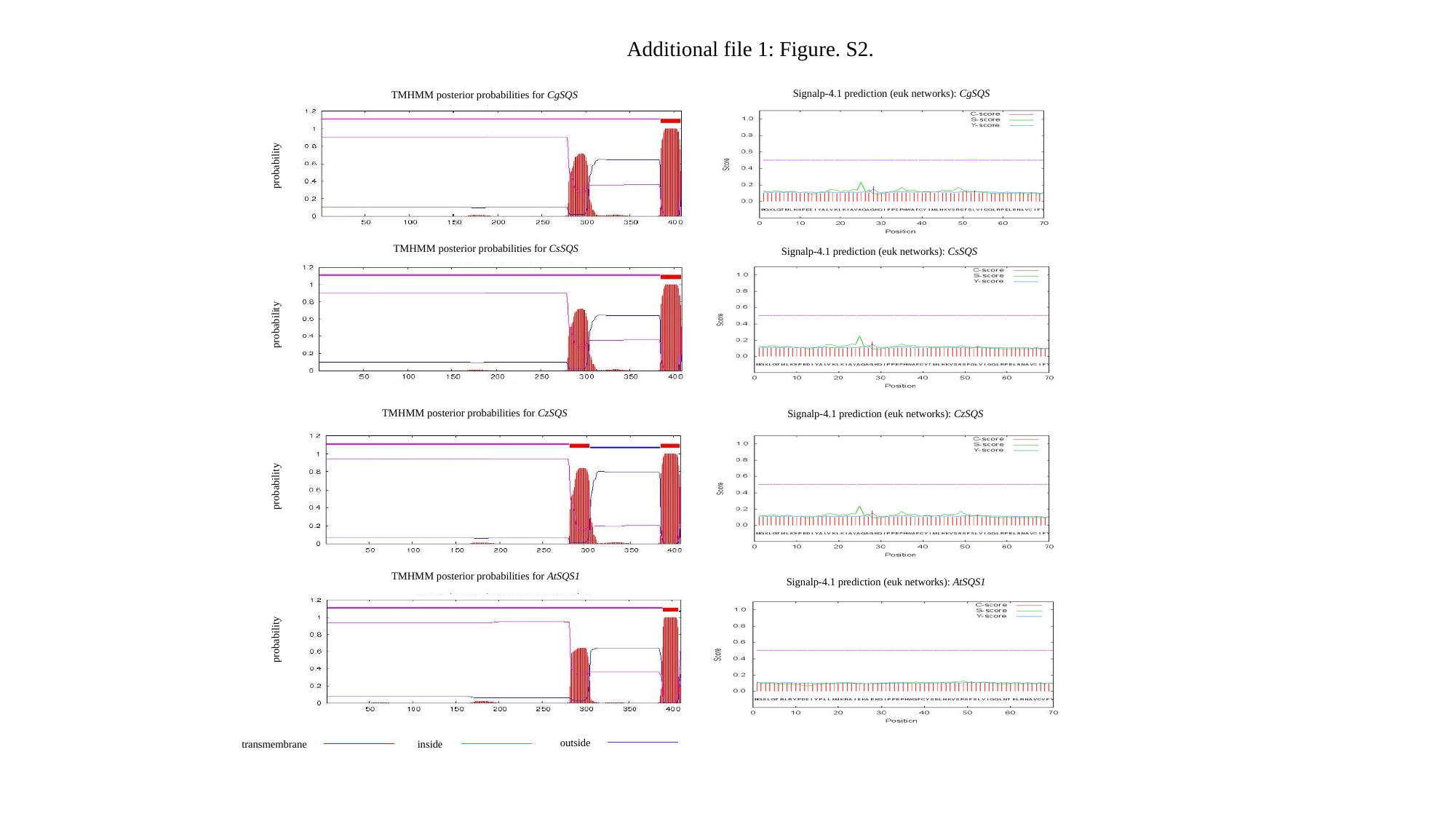

Additional file 1: Figure. S2.
Signalp-4.1 prediction (euk networks): CgSQS
TMHMM posterior probabilities for CgSQS
probability
TMHMM posterior probabilities for CsSQS
Signalp-4.1 prediction (euk networks): CsSQS
probability
TMHMM posterior probabilities for CzSQS
Signalp-4.1 prediction (euk networks): CzSQS
probability
TMHMM posterior probabilities for AtSQS1
Signalp-4.1 prediction (euk networks): AtSQS1
probability
outside
transmembrane
inside

## Slide 4
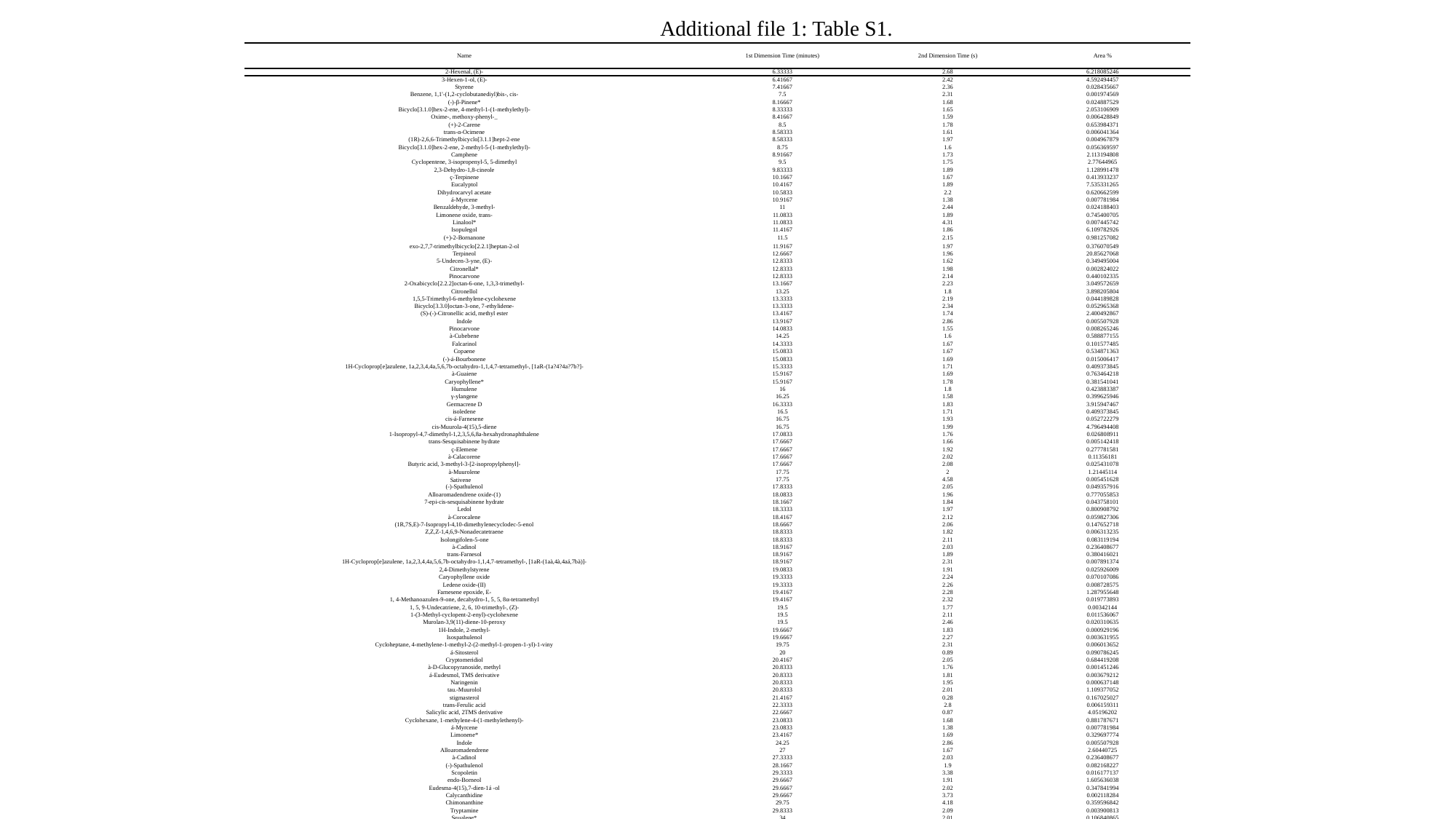

Additional file 1: Table S1.
| Name | 1st Dimension Time (minutes) | 2nd Dimension Time (s) | Area % |
| --- | --- | --- | --- |
| 2-Hexenal, (E)- | 6.33333 | 2.68 | 6.218085246 |
| 3-Hexen-1-ol, (E)- | 6.41667 | 2.42 | 4.592494457 |
| Styrene | 7.41667 | 2.36 | 0.028435667 |
| Benzene, 1,1'-(1,2-cyclobutanediyl)bis-, cis- | 7.5 | 2.31 | 0.001974569 |
| (-)-β-Pinene\* | 8.16667 | 1.68 | 0.024887529 |
| Bicyclo[3.1.0]hex-2-ene, 4-methyl-1-(1-methylethyl)- | 8.33333 | 1.65 | 2.053106909 |
| Oxime-, methoxy-phenyl-\_ | 8.41667 | 1.59 | 0.006428849 |
| (+)-2-Carene | 8.5 | 1.78 | 0.653984371 |
| trans-α-Ocimene | 8.58333 | 1.61 | 0.006041364 |
| (1R)-2,6,6-Trimethylbicyclo[3.1.1]hept-2-ene | 8.58333 | 1.97 | 0.004967879 |
| Bicyclo[3.1.0]hex-2-ene, 2-methyl-5-(1-methylethyl)- | 8.75 | 1.6 | 0.056369597 |
| Camphene | 8.91667 | 1.73 | 2.113194808 |
| Cyclopentene, 3-isopropenyl-5, 5-dimethyl | 9.5 | 1.75 | 2.77644965 |
| 2,3-Dehydro-1,8-cineole | 9.83333 | 1.89 | 1.128991478 |
| ç-Terpinene | 10.1667 | 1.67 | 0.413933237 |
| Eucalyptol | 10.4167 | 1.89 | 7.535331265 |
| Dihydrocarvyl acetate | 10.5833 | 2.2 | 0.620662599 |
| á-Myrcene | 10.9167 | 1.38 | 0.007781984 |
| Benzaldehyde, 3-methyl- | 11 | 2.44 | 0.024188403 |
| Limonene oxide, trans- | 11.0833 | 1.89 | 0.745400705 |
| Linalool\* | 11.0833 | 4.31 | 0.007445742 |
| Isopulegol | 11.4167 | 1.86 | 6.109782926 |
| (+)-2-Bornanone | 11.5 | 2.15 | 0.981257082 |
| exo-2,7,7-trimethylbicyclo[2.2.1]heptan-2-ol | 11.9167 | 1.97 | 0.376070549 |
| Terpineol | 12.6667 | 1.96 | 20.85627068 |
| 5-Undecen-3-yne, (E)- | 12.8333 | 1.62 | 0.349495004 |
| Citronellal\* | 12.8333 | 1.98 | 0.002824022 |
| Pinocarvone | 12.8333 | 2.14 | 0.440102335 |
| 2-Oxabicyclo[2.2.2]octan-6-one, 1,3,3-trimethyl- | 13.1667 | 2.23 | 3.049572659 |
| Citronellol | 13.25 | 1.8 | 3.898205804 |
| 1,5,5-Trimethyl-6-methylene-cyclohexene | 13.3333 | 2.19 | 0.044189828 |
| Bicyclo[3.3.0]octan-3-one, 7-ethylidene- | 13.3333 | 2.34 | 0.052965368 |
| (S)-(-)-Citronellic acid, methyl ester | 13.4167 | 1.74 | 2.400492867 |
| Indole | 13.9167 | 2.86 | 0.005507928 |
| Pinocarvone | 14.0833 | 1.55 | 0.008265246 |
| à-Cubebene | 14.25 | 1.6 | 0.588877155 |
| Falcarinol | 14.3333 | 1.67 | 0.101577485 |
| Copaene | 15.0833 | 1.67 | 0.534871363 |
| (-)-á-Bourbonene | 15.0833 | 1.69 | 0.015006417 |
| 1H-Cycloprop[e]azulene, 1a,2,3,4,4a,5,6,7b-octahydro-1,1,4,7-tetramethyl-, [1aR-(1a?4?4a?7b?]- | 15.3333 | 1.71 | 0.409373845 |
| à-Guaiene | 15.9167 | 1.69 | 0.763464218 |
| Caryophyllene\* | 15.9167 | 1.78 | 0.381541041 |
| Humulene | 16 | 1.8 | 0.423883387 |
| γ-ylangene | 16.25 | 1.58 | 0.399625946 |
| Germacrene D | 16.3333 | 1.83 | 3.915947467 |
| isoledene | 16.5 | 1.71 | 0.409373845 |
| cis-á-Farnesene | 16.75 | 1.93 | 0.052722279 |
| cis-Muurola-4(15),5-diene | 16.75 | 1.99 | 4.796494408 |
| 1-Isopropyl-4,7-dimethyl-1,2,3,5,6,8a-hexahydronaphthalene | 17.0833 | 1.76 | 0.026808911 |
| trans-Sesquisabinene hydrate | 17.6667 | 1.66 | 0.005142418 |
| ç-Elemene | 17.6667 | 1.92 | 0.277781581 |
| à-Calacorene | 17.6667 | 2.02 | 0.11356181 |
| Butyric acid, 3-methyl-3-[2-isopropylphenyl]- | 17.6667 | 2.08 | 0.025431078 |
| à-Muurolene | 17.75 | 2 | 1.21445114 |
| Sativene | 17.75 | 4.58 | 0.005451628 |
| (-)-Spathulenol | 17.8333 | 2.05 | 0.049357916 |
| Alloaromadendrene oxide-(1) | 18.0833 | 1.96 | 0.777055853 |
| 7-epi-cis-sesquisabinene hydrate | 18.1667 | 1.84 | 0.043758101 |
| Ledol | 18.3333 | 1.97 | 0.800908792 |
| à-Corocalene | 18.4167 | 2.12 | 0.059827306 |
| (1R,7S,E)-7-Isopropyl-4,10-dimethylenecyclodec-5-enol | 18.6667 | 2.06 | 0.147652718 |
| Z,Z,Z-1,4,6,9-Nonadecatetraene | 18.8333 | 1.82 | 0.006313235 |
| Isolongifolen-5-one | 18.8333 | 2.11 | 0.083119194 |
| à-Cadinol | 18.9167 | 2.03 | 0.236408677 |
| trans-Farnesol | 18.9167 | 1.89 | 0.380416021 |
| 1H-Cycloprop[e]azulene, 1a,2,3,4,4a,5,6,7b-octahydro-1,1,4,7-tetramethyl-, [1aR-(1aà,4à,4aá,7bà)]- | 18.9167 | 2.31 | 0.007891374 |
| 2,4-Dimethylstyrene | 19.0833 | 1.91 | 0.025926009 |
| Caryophyllene oxide | 19.3333 | 2.24 | 0.070107086 |
| Ledene oxide-(II) | 19.3333 | 2.26 | 0.008728575 |
| Farnesene epoxide, E- | 19.4167 | 2.28 | 1.287955648 |
| 1, 4-Methanoazulen-9-one, decahydro-1, 5, 5, 8α-tetramethyl | 19.4167 | 2.32 | 0.019773893 |
| 1, 5, 9-Undecatriene, 2, 6, 10-trimethyl-, (Z)- | 19.5 | 1.77 | 0.00342144 |
| 1-(3-Methyl-cyclopent-2-enyl)-cyclohexene | 19.5 | 2.11 | 0.011536067 |
| Murolan-3,9(11)-diene-10-peroxy | 19.5 | 2.46 | 0.020310635 |
| 1H-Indole, 2-methyl- | 19.6667 | 1.83 | 0.000929196 |
| Isospathulenol | 19.6667 | 2.27 | 0.003631955 |
| Cycloheptane, 4-methylene-1-methyl-2-(2-methyl-1-propen-1-yl)-1-viny | 19.75 | 2.31 | 0.006013652 |
| á-Sitosterol | 20 | 0.89 | 0.090786245 |
| Cryptomeridiol | 20.4167 | 2.05 | 0.684419208 |
| à-D-Glucopyranoside, methyl | 20.8333 | 1.76 | 0.001451246 |
| á-Eudesmol, TMS derivative | 20.8333 | 1.81 | 0.003679212 |
| Naringenin | 20.8333 | 1.95 | 0.000637148 |
| tau.-Muurolol | 20.8333 | 2.01 | 1.109377052 |
| stigmasterol | 21.4167 | 0.28 | 0.167025027 |
| trans-Ferulic acid | 22.3333 | 2.8 | 0.006159311 |
| Salicylic acid, 2TMS derivative | 22.6667 | 0.87 | 4.05196202 |
| Cyclohexane, 1-methylene-4-(1-methylethenyl)- | 23.0833 | 1.68 | 0.881787671 |
| á-Myrcene | 23.0833 | 1.38 | 0.007781984 |
| Limonene\* | 23.4167 | 1.69 | 0.329697774 |
| Indole | 24.25 | 2.86 | 0.005507928 |
| Alloaromadendrene | 27 | 1.67 | 2.60440725 |
| à-Cadinol | 27.3333 | 2.03 | 0.236408677 |
| (-)-Spathulenol | 28.1667 | 1.9 | 0.082168227 |
| Scopoletin | 29.3333 | 3.38 | 0.016177137 |
| endo-Borneol | 29.6667 | 1.91 | 1.605636038 |
| Eudesma-4(15),7-dien-1á -ol | 29.6667 | 2.02 | 0.347841994 |
| Calycanthidine | 29.6667 | 3.73 | 0.002118284 |
| Chimonanthine | 29.75 | 4.18 | 0.359596842 |
| Tryptamine | 29.8333 | 2.09 | 0.003900813 |
| Squalene\* | 34 | 2.01 | 0.106840865 |
| Coumarin, 6-benzyloxy-3,4-dihydro-4,4-dimethyl-7-nitro- | 34 | 1.89 | 0.001871402 |
| (-)-Aristolene | 34.8333 | 1.8 | 1.181772098 |
| á Carotene | 34.8333 | 4.55 | 0.003399173 |

## Slide 5
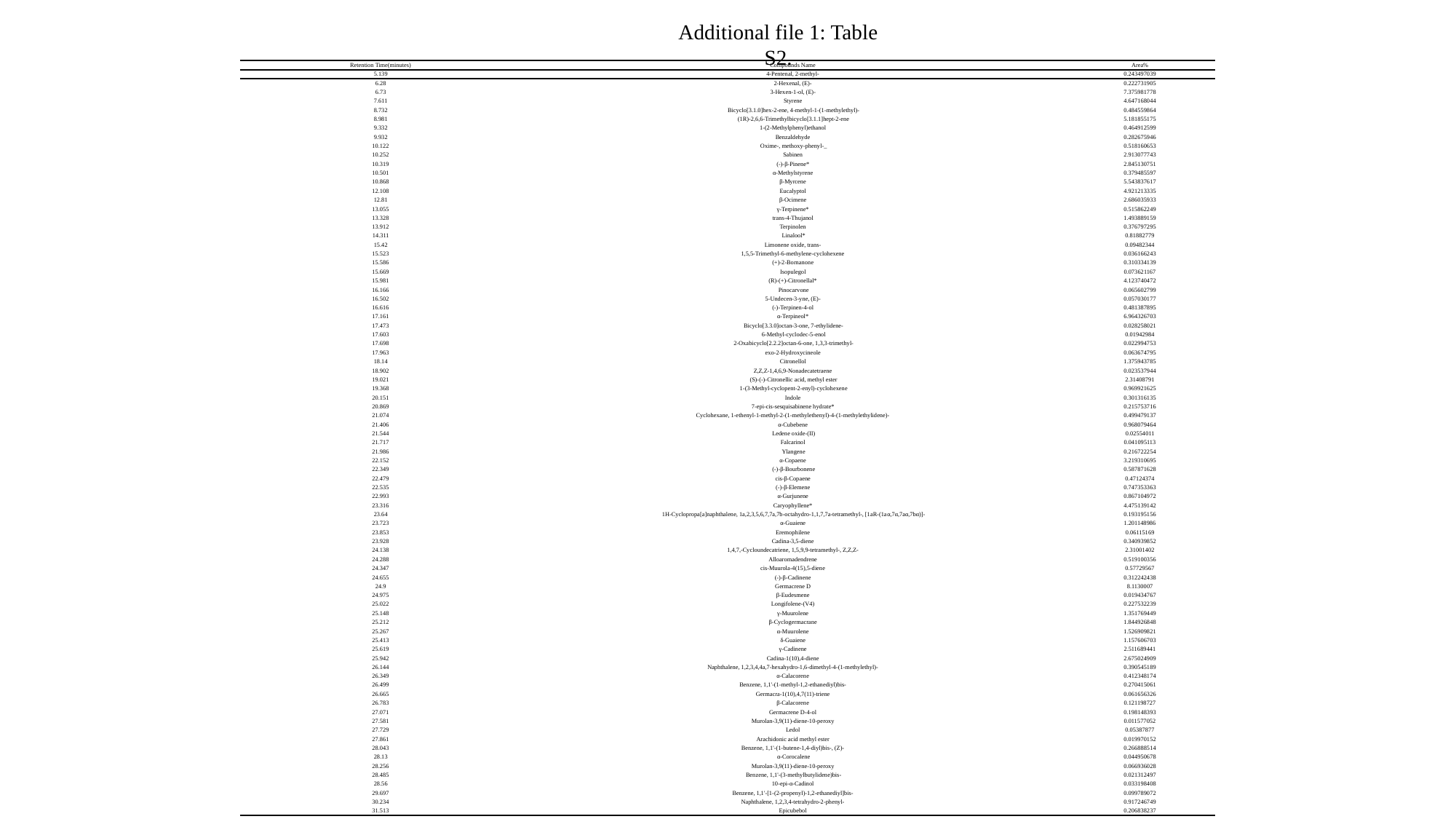

Additional file 1: Table S2.
| Retention Time(minutes) | Compounds Name | Area% |
| --- | --- | --- |
| 5.139 | 4-Pentenal, 2-methyl- | 0.243497039 |
| 6.28 | 2-Hexenal, (E)- | 0.222731905 |
| 6.73 | 3-Hexen-1-ol, (E)- | 7.375981778 |
| 7.611 | Styrene | 4.647168044 |
| 8.732 | Bicyclo[3.1.0]hex-2-ene, 4-methyl-1-(1-methylethyl)- | 0.484559864 |
| 8.981 | (1R)-2,6,6-Trimethylbicyclo[3.1.1]hept-2-ene | 5.181855175 |
| 9.332 | 1-(2-Methylphenyl)ethanol | 0.464912599 |
| 9.932 | Benzaldehyde | 0.282675946 |
| 10.122 | Oxime-, methoxy-phenyl-\_ | 0.518160653 |
| 10.252 | Sabinen | 2.913077743 |
| 10.319 | (-)-β-Pinene\* | 2.845130751 |
| 10.501 | α-Methylstyrene | 0.379485597 |
| 10.868 | β-Myrcene | 5.543837617 |
| 12.108 | Eucalyptol | 4.921213335 |
| 12.81 | β-Ocimene | 2.686035933 |
| 13.055 | γ-Terpinene\* | 0.515862249 |
| 13.328 | trans-4-Thujanol | 1.493889159 |
| 13.912 | Terpinolen | 0.376797295 |
| 14.311 | Linalool\* | 0.81882779 |
| 15.42 | Limonene oxide, trans- | 0.09482344 |
| 15.523 | 1,5,5-Trimethyl-6-methylene-cyclohexene | 0.036166243 |
| 15.586 | (+)-2-Bornanone | 0.310334139 |
| 15.669 | Isopulegol | 0.073621167 |
| 15.981 | (R)-(+)-Citronellal\* | 4.123740472 |
| 16.166 | Pinocarvone | 0.065602799 |
| 16.502 | 5-Undecen-3-yne, (E)- | 0.057030177 |
| 16.616 | (-)-Terpinen-4-ol | 0.481387895 |
| 17.161 | α-Terpineol\* | 6.964326703 |
| 17.473 | Bicyclo[3.3.0]octan-3-one, 7-ethylidene- | 0.028258021 |
| 17.603 | 6-Methyl-cyclodec-5-enol | 0.01942984 |
| 17.698 | 2-Oxabicyclo[2.2.2]octan-6-one, 1,3,3-trimethyl- | 0.022994753 |
| 17.963 | exo-2-Hydroxycineole | 0.063674795 |
| 18.14 | Citronellol | 1.375943785 |
| 18.902 | Z,Z,Z-1,4,6,9-Nonadecatetraene | 0.023537944 |
| 19.021 | (S)-(-)-Citronellic acid, methyl ester | 2.31408791 |
| 19.368 | 1-(3-Methyl-cyclopent-2-enyl)-cyclohexene | 0.969921625 |
| 20.151 | Indole | 0.301316135 |
| 20.869 | 7-epi-cis-sesquisabinene hydrate\* | 0.215753716 |
| 21.074 | Cyclohexane, 1-ethenyl-1-methyl-2-(1-methylethenyl)-4-(1-methylethylidene)- | 0.499479137 |
| 21.406 | α-Cubebene | 0.968079464 |
| 21.544 | Ledene oxide-(II) | 0.02554011 |
| 21.717 | Falcarinol | 0.041095113 |
| 21.986 | Ylangene | 0.216722254 |
| 22.152 | α-Copaene | 3.219310695 |
| 22.349 | (-)-β-Bourbonene | 0.587871628 |
| 22.479 | cis-β-Copaene | 0.47124374 |
| 22.535 | (-)-β-Elemene | 0.747353363 |
| 22.993 | α-Gurjunene | 0.867104972 |
| 23.316 | Caryophyllene\* | 4.475139142 |
| 23.64 | 1H-Cyclopropa[a]naphthalene, 1a,2,3,5,6,7,7a,7b-octahydro-1,1,7,7a-tetramethyl-, [1aR-(1aα,7α,7aα,7bα)]- | 0.193195156 |
| 23.723 | α-Guaiene | 1.201148986 |
| 23.853 | Eremophilene | 0.06115169 |
| 23.928 | Cadina-3,5-diene | 0.340939852 |
| 24.138 | 1,4,7,-Cycloundecatriene, 1,5,9,9-tetramethyl-, Z,Z,Z- | 2.31001402 |
| 24.288 | Alloaromadendrene | 0.519100356 |
| 24.347 | cis-Muurola-4(15),5-diene | 0.57729567 |
| 24.655 | (-)-β-Cadinene | 0.312242438 |
| 24.9 | Germacrene D | 8.1130007 |
| 24.975 | β-Eudesmene | 0.019434767 |
| 25.022 | Longifolene-(V4) | 0.227532239 |
| 25.148 | γ-Muurolene | 1.351769449 |
| 25.212 | β-Cyclogermacrane | 1.844926848 |
| 25.267 | α-Muurolene | 1.526909821 |
| 25.413 | δ-Guaiene | 1.157606703 |
| 25.619 | γ-Cadinene | 2.511689441 |
| 25.942 | Cadina-1(10),4-diene | 2.675024909 |
| 26.144 | Naphthalene, 1,2,3,4,4a,7-hexahydro-1,6-dimethyl-4-(1-methylethyl)- | 0.390545189 |
| 26.349 | α-Calacorene | 0.412348174 |
| 26.499 | Benzene, 1,1'-(1-methyl-1,2-ethanediyl)bis- | 0.270415061 |
| 26.665 | Germacra-1(10),4,7(11)-triene | 0.061656326 |
| 26.783 | β-Calacorene | 0.121198727 |
| 27.071 | Germacrene D-4-ol | 0.198148393 |
| 27.581 | Murolan-3,9(11)-diene-10-peroxy | 0.011577052 |
| 27.729 | Ledol | 0.05387877 |
| 27.861 | Arachidonic acid methyl ester | 0.019970152 |
| 28.043 | Benzene, 1,1'-(1-butene-1,4-diyl)bis-, (Z)- | 0.266888514 |
| 28.13 | α-Corocalene | 0.044950678 |
| 28.256 | Murolan-3,9(11)-diene-10-peroxy | 0.066936028 |
| 28.485 | Benzene, 1,1'-(3-methylbutylidene)bis- | 0.021312497 |
| 28.56 | 10-epi-α-Cadinol | 0.033198408 |
| 29.697 | Benzene, 1,1'-[1-(2-propenyl)-1,2-ethanediyl]bis- | 0.099789072 |
| 30.234 | Naphthalene, 1,2,3,4-tetrahydro-2-phenyl- | 0.917246749 |
| 31.513 | Epicubebol | 0.206838237 |

## Slide 6
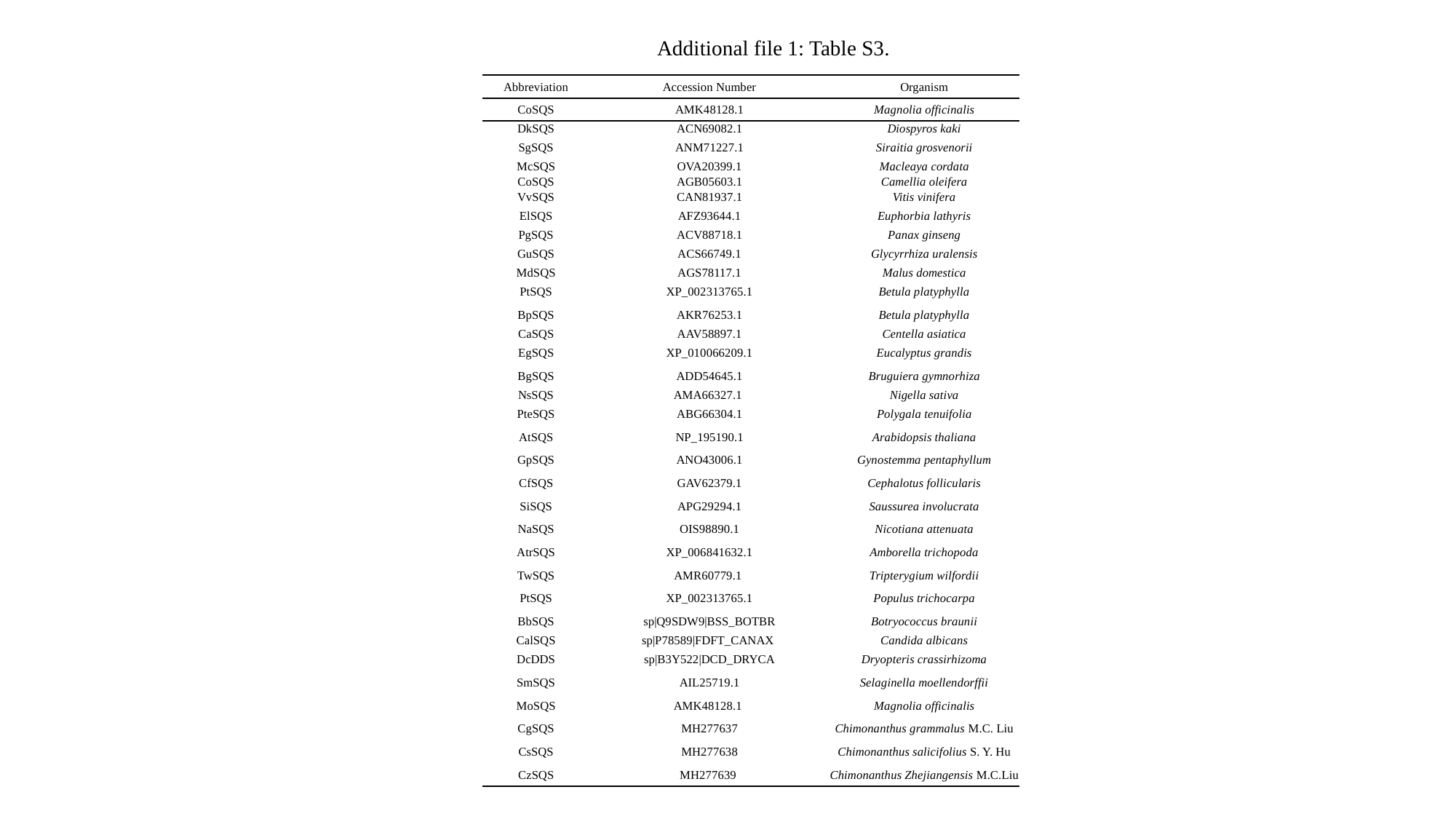

Additional file 1: Table S3.
| Abbreviation | Accession Number | Organism |
| --- | --- | --- |
| CoSQS | AMK48128.1 | Magnolia officinalis |
| DkSQS | ACN69082.1 | Diospyros kaki |
| SgSQS | ANM71227.1 | Siraitia grosvenorii |
| McSQS | OVA20399.1 | Macleaya cordata |
| CoSQS | AGB05603.1 | Camellia oleifera |
| VvSQS | CAN81937.1 | Vitis vinifera |
| ElSQS | AFZ93644.1 | Euphorbia lathyris |
| PgSQS | ACV88718.1 | Panax ginseng |
| GuSQS | ACS66749.1 | Glycyrrhiza uralensis |
| MdSQS | AGS78117.1 | Malus domestica |
| PtSQS | XP\_002313765.1 | Betula platyphylla |
| BpSQS | AKR76253.1 | Betula platyphylla |
| CaSQS | AAV58897.1 | Centella asiatica |
| EgSQS | XP\_010066209.1 | Eucalyptus grandis |
| BgSQS | ADD54645.1 | Bruguiera gymnorhiza |
| NsSQS | AMA66327.1 | Nigella sativa |
| PteSQS | ABG66304.1 | Polygala tenuifolia |
| AtSQS | NP\_195190.1 | Arabidopsis thaliana |
| GpSQS | ANO43006.1 | Gynostemma pentaphyllum |
| CfSQS | GAV62379.1 | Cephalotus follicularis |
| SiSQS | APG29294.1 | Saussurea involucrata |
| NaSQS | OIS98890.1 | Nicotiana attenuata |
| AtrSQS | XP\_006841632.1 | Amborella trichopoda |
| TwSQS | AMR60779.1 | Tripterygium wilfordii |
| PtSQS | XP\_002313765.1 | Populus trichocarpa |
| BbSQS | sp|Q9SDW9|BSS\_BOTBR | Botryococcus braunii |
| CalSQS | sp|P78589|FDFT\_CANAX | Candida albicans |
| DcDDS | sp|B3Y522|DCD\_DRYCA | Dryopteris crassirhizoma |
| SmSQS | AIL25719.1 | Selaginella moellendorffii |
| MoSQS | AMK48128.1 | Magnolia officinalis |
| CgSQS | MH277637 | Chimonanthus grammalus M.C. Liu |
| CsSQS | MH277638 | Chimonanthus salicifolius S. Y. Hu |
| CzSQS | MH277639 | Chimonanthus Zhejiangensis M.C.Liu |
